# Revisiting hereditary tyrosinemia Type 1—spectrum of radiological findings

**DOI:** 10.1259/bjrcr.20180001

**Published:** 2018-11-07

**Authors:** Sana Shaikh, Asma Qureshi, Syed Mohammad faiq

**Affiliations:** 1 Department of Radiology, Sindh Institute of Urology and Transplantation, Karachi, Pakistan

## Abstract

Tyrosinemia is a rare metabolic disease showing autosomal recessive inheritance associated with a deficiency of the enzyme fumarylacetoacetate hydrolase. Absence of this enzyme results in the accumulation of succinylacetone in the tissues which predominantly results in liver injury, renal tubular damage, and neurological manifestation resembling porphyrias. The complications that can develop without appropriate treatment include renal tubular dysfunction, growth failure, rickets, neurological crises, hepatomegaly, and possible hepatocellular carcinoma. We describe a case of 18-month-old child who presents with fever and gradually progressive abdominal distension. Laboratory and radiological investigations were done that lead to the diagnosis of this rare entity.

## Summary

Tyrosinemia Type I, also called as hepatorenal tyrosinemia is an autosomal recessive disease caused by defect in the enzyme involved in the degradation of tyrosine. This deficiency leads to an accumulation of substances that cause cellular damage. Clinical symptoms usually begin before 2 years of age. The majority of children present before the age of 6 months with evidence of acute liver failure and renal dysfunction. Neurological manifestations occur as painful episodes affecting extremity and/or abdominal function, along with hypertension and hyponatremia, and may result in respiratory failure and death. In hepatorenal tyrosinemia, liver is mostly affected. The frequent reason of presentation is acute hepatic failure. Majority of deaths are caused by liver failure and hepatocellular carcinoma. This case describes salient features of hereditary tyrosinemia Type I and also emphasizes on the importance of imaging in the diagnosis of this rare metabolic disorder and the differential diagnosis.

## Case presentation

An 18-month-old child who came with the complaints of intermittent spiking fever, constipation, and abdominal distension since 1 month after birth. The distension of abdomen was gradual in onset. His birth history and immunization investigations were unremarkable. One noticeable element in his family history was that his sister suffered from similar kind of illness but the parents did not seek any medical attention and she died at 2 years of age.

## Investigations

**Figure 1.**
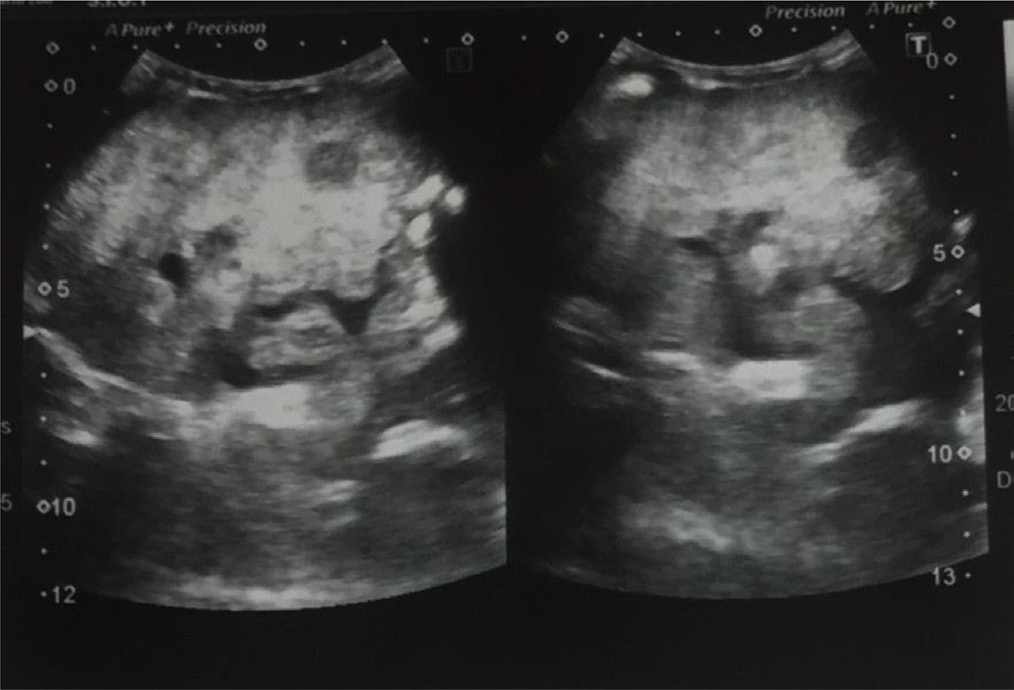
Ultrasound abdomen shows cirrhotic liver with irregular nodular margins and coarse texture. A small hypoechoic mass lesion is also evident within it.

**Figure 2.**
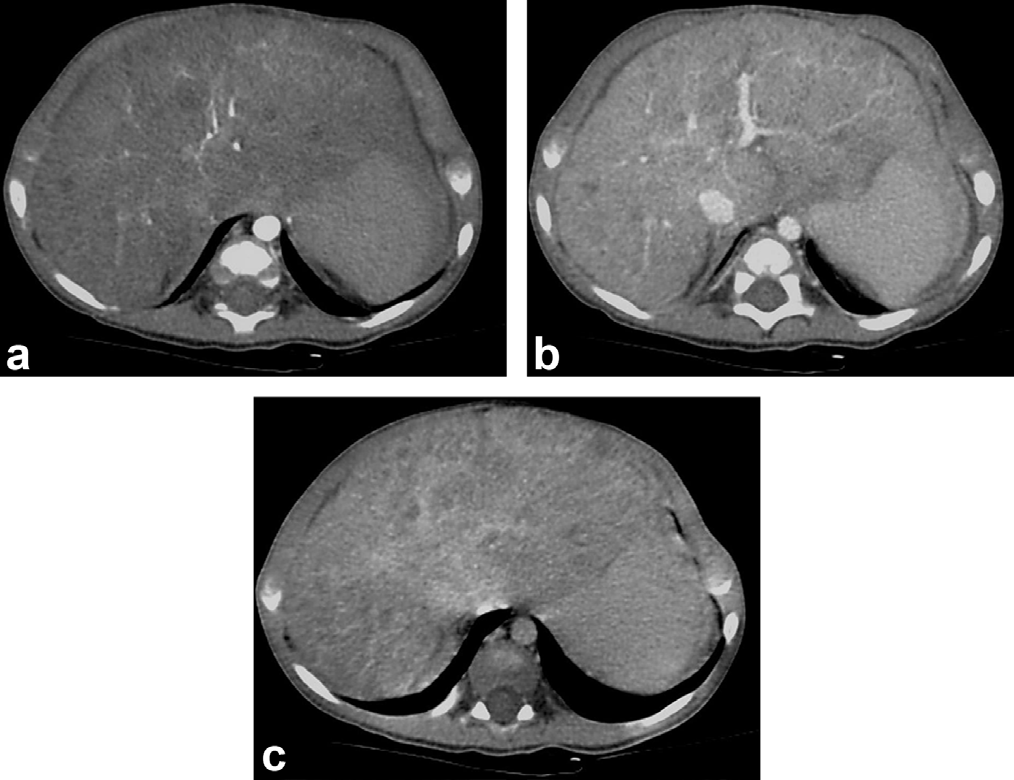
(a) Arterial phase of tri-phasic CT scan shows multiple non-enhancing nodules in liver. (b) Venous phase of tri-phasic CT scan. (c) Delayed images of tri-phasic CT scan.

**Figure 3.**
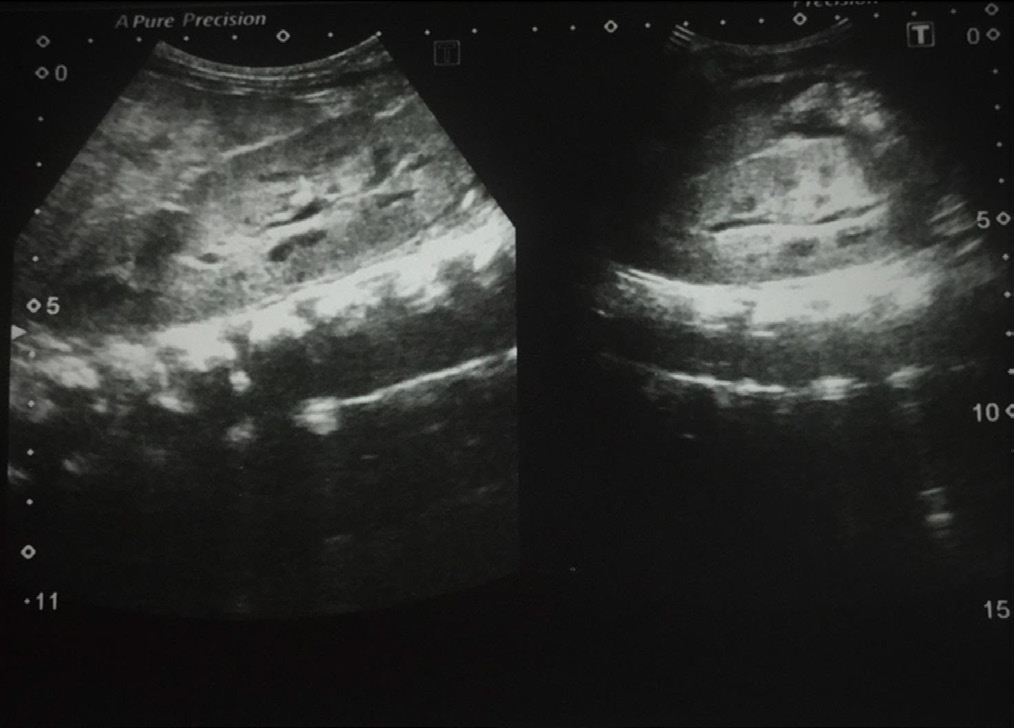
Ultrasound abdomen demonstrates bilateral enlarged echogenic kidneys.

**Figure 4.**
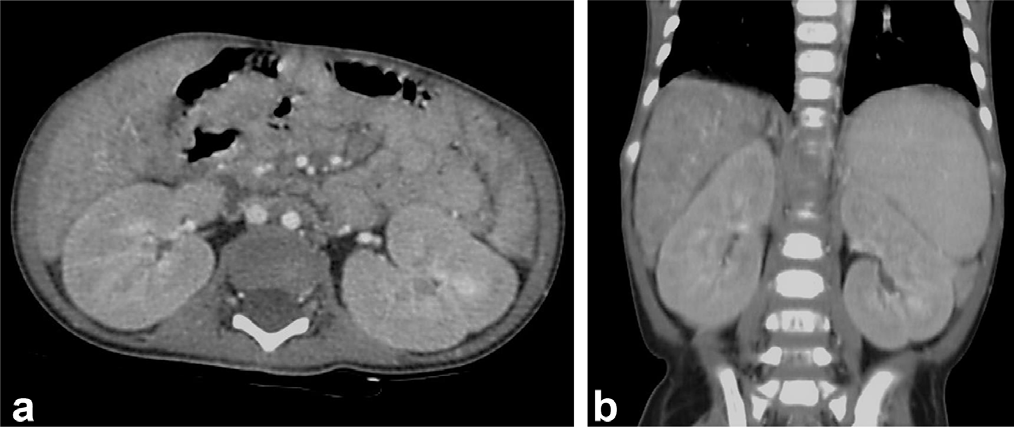
(a) CT scan, post-contrast, axial images demonstrate hypodense kidneys with indistinct corticomedullary distinction. (b) CT scan, coronal images shows large-sized hypodense kidneys.

**Figure 5.**
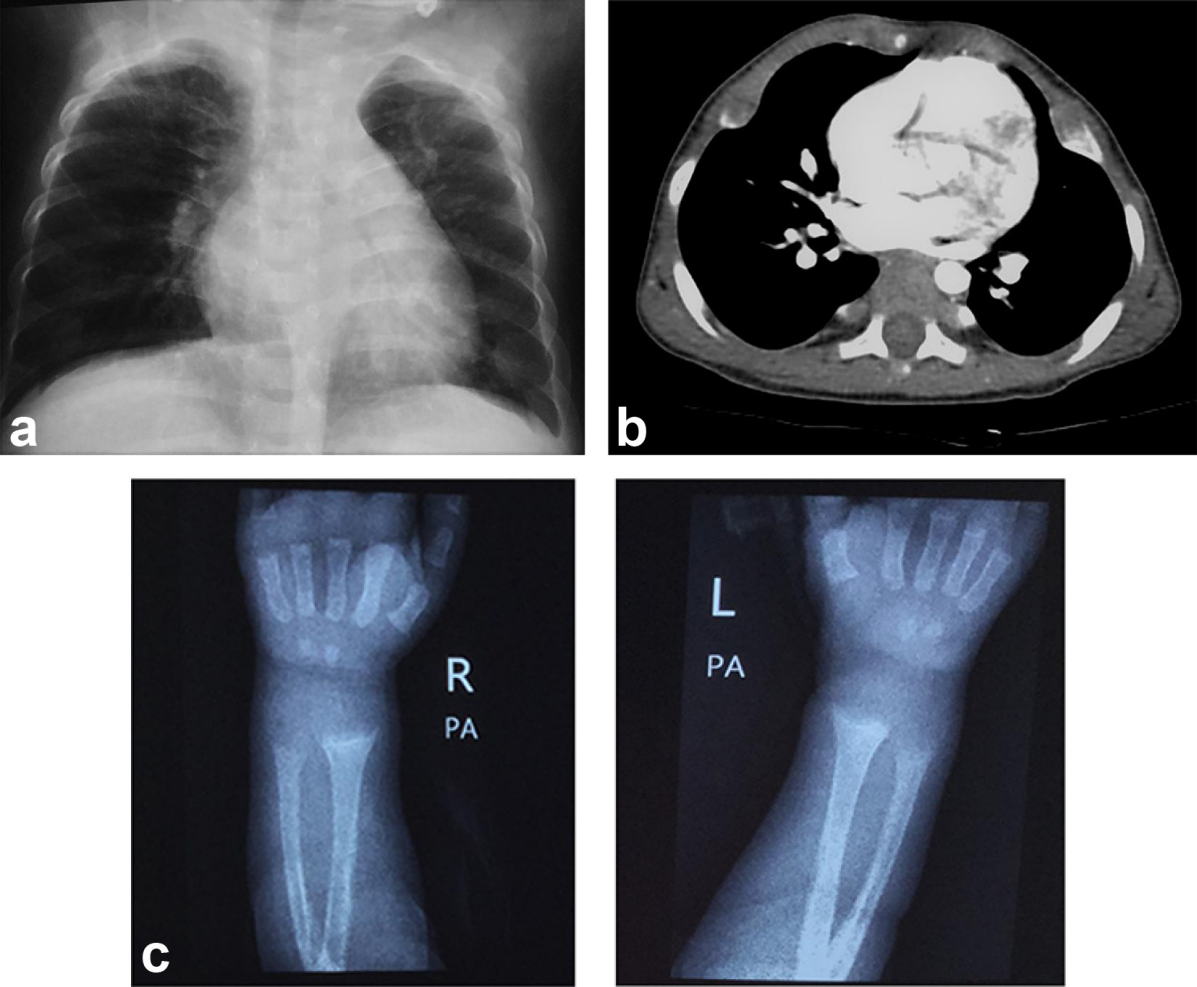
(a) Chest radiograph anteroposterior view, expansion of the anterior ends of ribs is clearly evident, particularly in the left lower ribs. (b) CT scan chest axial view also shows expansion of anterior ends of ribs consistent with rachitic rosary.(c) X-ray of both wrist joints. There is loss of provisional zone of calcification with widening of growth plate. Metaphysis appears flared and fraying is also evident. All these findings point towards rickets.

His laboratory investigations showed anemia with hemoglobin of 8.0 gm dl^−1^, leukocytosis with TLC count of 1,2000. Liver function tests demonstrated markedly raised alkaline phosphatase of 2117 U l^−1^, SGOT of 121 U l^−1^ and γ GT of 301 U l^−1^. Total bilirubin was also raised. Prothrombin time and INR were also markedly deranged. His workup for TORCH was also unremarkable. On clinical examination, patient was below fifth percentile of height and weight. He is jaundiced and distension of abdomen was also evident. On palpation, liver and spleen appeared to be borderline enlarged. Based on the aforementioned history, clinical examination and serum alpha-feto protein levels were ordered which turned out to be strikingly high with the level of 14,831. Ultrasound abdomen was then done that displayed coarse altered texture of liver parenchyma with irregular and nodular appearance; multiple small hypoechoic avascular mass lesions were also noted (Figure 1). These correspond to non-enhancing mass lesions in post-contrast CT scan. Typical enhancement pattern of hepatocellular carcinoma was not seen, so these were diagnosed as likely regenerating or dysplastic nodules in the background of cirrhotic liver (Figure 2a–c . Portal vein was patent and splenomegaly was also noticed. Signs of portal hypertension were not there. Both kidneys were also enlarged and echogenic on ultrasound (Figure 3) These findings ae equivalent to the imaging appearance of kidneys on post-contrast CT scan which show poor enhancement with loss of cortico medullary distinction Figure 4a,b). In addition there was expansion of the anterior ribs at the costochondral junction indicating rachitic rosary; it was also visible on frontal chest X-ray done as a part of routine workup. X-ray of both wrist joints further confirmed the additional findings of rickets (Figure 5a–c).

## Diagnosis and differential diagnosis

These warning signs of cirrhotic liver with deranged liver function tests lead us to think about metabolic disorders like glycogen storage disease and Wilson’s disease. Nonetheless, combining the additional imaging findings of enlarged kidneys and rickets strongly indicate the possibility of hereditary tyrosinemia Type 1. To substantiate the diagnosis, urinary organic acid were then requested which determined high level of succinylacetone. The diagnosis of hereditary tyrosinemia Type 1 was hence confirmed.

## Treatment

Treatment plan was to counsel patient’s parents about dietary restriction of phenylalanine and tyrosine and subsequent liver transplantation.

## Outcome and follow up

Patient had been worked up for liver transplantation.

## Discussion

Tyrosinemia Type I is a rare inherited metabolic disorder with a worldwide incidence of 1:100,000–1:120,000 with highest incidence in Scandinavia and Quebec, where the incidence is 1:20,000.^1^

Clinical symptoms usually begin before 2 years of age. The majority of children present before the age of 6 months with evidence of acute liver failure and renal dysfunction. It is one of the few metabolic disorders with characteristic odor, which is described as “boiled cabbage” or “rotten mushroom” smell.^2^ This feature was not observed in our patient. According to age at start of symptoms it is classified as acute (<6 months), subacute (6–12 months), and chronic (>1 year) forms.^3^ α feto protein level was strikingly raised in our patient and was clinically thought to have hepatoblastoma or hepatocellular carcinoma; however, imaging refuted this suspicion. It is known that alfa feto protein is a sensitive but not a specific marker for hepatocellular carcinoma.^4^ The most helpful test is to test succinylacetone levels in urine, plasma or dried blood spot.^3^ Screening the newborns can lead to early diagnosis of the disease.^5^

Liver changes are most common in tyrosinemia which includes changes of cirrhosis with regenerative nodules or fatty infiltration or even space occupying lesion like hepatocellular carcinoma.^6^ Imaging is important in the evaluation of hepatorenal tyrosinemia, especially CT and/or MRI abdomen with contrast, plays a key role in the diagnosis of hepatocellular carcinomaHCC.^7^ Renal enlargement and nephrocalcinosis are usually seen in these patients.^8^ Hypophosphatemic rickets is frequently found secondary to renal involvement.^9^ In our patient, although renal failure was not present to date but imaging did manifest renal involvement and the radiographs exhibited changes of rickets. Another imaging modality that is worth mentioning here is MRI brain. Although there is scarcity of literature to describe findings on MRI brain, one case report did outline some findings that could be found in chronic hereditary tyrosinemia. On *T*_2_ weighted high signals can be seen in bilateral globus pallidus.^10^ In our case MRI brain was not done because the diagnosis had already been made and patient did not have neurological symptoms so far. Other organs involved in tyrosinemic patients include the pancreas. Pancreatic atrophy along with echogenic parenchyma and hypoglycemia has been reported.^11^

Thus the differential diagnosis includes Wilson’s disease, mitochondrial disorders, and disorders of carbohydrate metabolism. Treatment includes nitisinone and a protein restricted diet in addition to long-term management by a specialist. Liver transplantation is another option for severe disease or complications of this disorder.^12^

## Learning points

1. Hereditary tyrosinemia Type 1, although being a very rare disease possesses typical imaging spectrum. The knowledge of imaging features of this metabolic disease is very crucial in leading towards early and accurate diagnosis in those patients where the clinical sign and symptoms are equivocal.
2. The usefulness of imaging also lies in early diagnosis of hepatocellular carcinoma before the disease becomes metastatic.
3. As the clinical features closely resemble other metabolic diseases, index of suspicion of this disease should be kept high in a child if there is cirrhotic liver with or without hepatocellular carcinoma with noticeably raised alpha-fetoprotein levels.
